# Genome Sequences of Two Temperate Phages, ΦCB2047-A and ΦCB2047-C, Infecting *Sulfitobacter* sp. Strain 2047

**DOI:** 10.1128/genomeA.00108-14

**Published:** 2014-06-05

**Authors:** Nana Y. D. Ankrah, Charles R. Budinoff, William H. Wilson, Steven W. Wilhelm, Alison Buchan

**Affiliations:** a Department of Microbiology, University of Tennessee, Knoxville, Tennessee, USA; b Bigelow Laboratory for Ocean Sciences, East Boothbay, Maine, USA

## Abstract

We announce the complete genome sequences of two temperate *Podoviridae*, *Sulfitobacter* phages ΦCB2047-A and ΦCB2047-C, which infect *Sulfitobacter* sp. strain 2047, a member of the *Roseobacter* clade. This is the first report of temperate podophage infecting members of the *Sulfitobacter* genus of the *Roseobacter* clade.

## GENOME ANNOUNCEMENT

We report here the genomes of two lysogenic *Podoviridae*, phages ΦCB2047-A and ΦCB2047-C, infecting *Sulfitobacter* sp. strain 2047, a member of the *Roseobacter* clade of marine bacteria. This is the first report of temperate *Podoviridae* infecting members of the *Sulfitobacter* genus of the *Roseobacter* clade. The two podophages were isolated from an induced algal bloom mesocosm study in Raunefjorden, Norway, using standard plaque assay techniques (1, 2), and were sequenced by the Broad Institute under the Gordon and Betty Moore Foundation’s Marine Phage, Virus, and Virome Sequencing Project. An average sequencing coverage of ≈30× was obtained for both phages. Genome annotations were done using the RAST annotation server (3) and tRNAscan-SE search server (4). Translated peptides from the phage genomes were used as BLASTp queries to the NCBI nonredundant protein sequence database to manually curate possible gene functions and identify the nearest phage or prophage relatives. The CoreGenesUniqueGenes (CGUG) genome analysis tool (5) was used to identify gene homologues and assign core genes that are shared with other closely related phages.

Phage ΦCB2047-A is 40,929 bp, with a G+C content of 58.8%. A total of 73 open reading frames (ORFs) were identified in phage ΦCB2047-A. Phage ΦCB2047-C is 40,931 bp, with a G+C content of 59%. A total of 73 ORFs were identified in phage ΦCB2047-C. Phages ΦCB2047-A and ΦCB2047-C are nearly identical at the nucleotide level, except for a ~2,000-bp region encoding a T5orf172 domain-containing protein (PF10544) and RusA-like endodeoxyribonuclease in ΦCB2047-A and five hypothetical proteins in ΦCB2047-C, where they share no sequence similarity. ΦCB2047-A and ΦCB2047-C share greatest sequence similarity to ΦEBPR podovirus 2, an uncultured phage from an enhanced biological phosphorus removal reactor (6). CGUG analysis identified 17 highly homologous genes (BLASTp threshold score, 85) between ΦCB2047-A and ΦCB2047-C and ΦEBPR podovirus 2. Both ΦCB2047-A and ΦCB2047-C have a DNA Bre-C like integrase to integrate in the host genome and lysis/lysozyme proteins with glycosyl hydrolase and peptidoglycan binding domains predicted to be involved in host cell lysis cells. Phages ΦCB2047-A and ΦCB2047-C also show relatedness to the temperate *Myxococcus* phage Mx8 (accession no. NC_003085), with protein homology existing within the terminase gene and several putative tail-fiber genes.

In contrast to other known roseophages, the genomes of ΦCB2047-A and ΦCB2047-C do not contain genes showing strong homology to currently described DNA polymerases, thymidylate synthases, ribonucleotide reductases, and deoxycytidine deaminases (7). The absence of well-characterized replication/nucleotide metabolism genes indicates that ΦCB2047-A and ΦCB2047-C may rely heavily on host resources for nucleotide production to generate new virions or possibly use novel replication and nucleotide metabolism proteins. Also absent from the genomes of ΦCB2047-A and ΦCB2047-C are homologs to known DNA methylases, which are frequently present in other temperate relatives (8), including ΦEBPR podovirus 2 (accession no. AEI70896.1). The genomes of ΦCB2047-A and ΦCB2047-C encode homing endonucleases (HNH_3 domain [Pfam13392]), which may be beneficial to the host and/or offer a competitive advantage to the phage by cleaving the DNA of other closely related competing phages during mixed infections (9).

### Nucleotide sequence accession numbers.

The whole-genome sequences of *Sulfitobacter* phages ΦCB2047-A and ΦCB2047-C were deposited in GenBank under the accession no. HQ332142 and HQ317384, respectively.
